# Clinicopathological and Genetic Characteristics of Patients of Different Ages with Diffuse Sclerosing Variant Papillary Thyroid Carcinoma

**DOI:** 10.3390/cancers15123101

**Published:** 2023-06-07

**Authors:** Soo-Young Kim, Su-Jin Shin, Dong-Gi Lee, Hyeok-Jun Yun, Seok-Mo Kim, Hojin Chang, Hang-Seok Chang, Hyunjung Shin, Yong-Sang Lee

**Affiliations:** 1 Department of Surgery, Ajou University School of Medicine, Suwon 16499, Republic of Korea; kimsuy@aumc.ac.kr; 2 Department of Pathology, Gangnam Severance Hospital, Yonsei University College of Medicine, Seoul 03722, Republic of Korea; charm@yuhs.ac; 3 Department of Psychiatry, Ajou University School of Medicine, Suwon 16499, Republic of Korea; donggi.lee@pennmedicine.upenn.edu; 4 Department of Biostatistics, Epidemiology and Informatics, Perelman School of Medicine, University of Pennsylvania, Philadelphia, PA 19104, USA; 5 Department of Surgery, Thyroid Cancer Center, Gangnam Severance Hospital, Institute of Refractory Thyroid Cancer, Yonsei University College of Medicine, Seoul 03722, Republic of Korea; gsyhj@yuhs.ac (H.-J.Y.); seokmokim@yuhs.ac (S.-M.K.); docjang@yuhs.ac (H.C.); surghsc@yuhs.ac (H.-S.C.); 6 Department of Industrial Engineering, Ajou University, Suwon 16499, Republic of Korea; 7 Department of Artificial Intelligence, Ajou University, Suwon 16499, Republic of Korea

**Keywords:** diffuse sclerosing variant papillary thyroid carcinoma, K-Ras, *BRAF*, *TERT*, thyroid malignancy, papillary thyroid cancer, gene

## Abstract

**Simple Summary:**

Diffuse sclerosing variant papillary thyroid carcinoma is a rare variant of papillary thyroid carcinoma that is most frequently observed in young patients with different clinical, pathological, and molecular profiles to classical PTC. Our findings revealed significant age-related differences in DSVPTC. DSVPTC was more aggressive in paediatric patients with a larger-sized tumour, more common multiplicity, and lateral neck metastasis. Despite being frequent and more aggressive in younger patients, DSVPTC also occurs in older patients with aggressive behaviour. Through targeted next-generation sequencing, we identified the *BRAF*, *KRAS*, and *TERT* mutations as the most important genes in DSVPTC with age-specific differences.

**Abstract:**

Diffuse sclerosing variant papillary thyroid carcinoma (DSVPTC) is commonly observed in young patients, with a median age at diagnosis in the third decade of life. Further, the risk of recurrence is higher for DSVPTC than for classical PTC. Therefore, this study aimed to describe the clinicopathological and genetic characteristics of patients of different ages with DSVPTC. We retrospectively reviewed 397 patients who underwent thyroidectomy for DSVPTC at Gangnam Severance Hospital, Yonsei University, from January 2005 to December 2017. The mean age at diagnosis was 36.7 ± 11.6 years, with most patients (163, 41.1%) aged 31–40 years. DSVPTC was predominant in women (276, 69.5%). We observed recurrence in 46 (11.6%) patients, with regional nodal recurrence being the most common type of recurrence (32 patients, 69.6%). The mean tumour size was larger in younger patients than in older patients. DSVPTC was more aggressive in paediatric patients with a larger-sized tumour, more common multiplicity, and lateral neck metastasis. Through random sampling, we selected 41 patients by age group and examined the mutations in 119 genes using next-generation sequencing. *BRAF*, *KRAS*, and *TERT* displayed relatively higher mutation rates than other genes. DSVPTC displays different clinical, pathological, and molecular profiles than classical PTC. The *BRAF*, *KRAS*, and *TERT* mutations are the most important, with age-specific differences.

## 1. Introduction

Papillary thyroid carcinoma (PTC) is the most frequent subtype among multiple thyroid malignancies, and its incidence is one of the highest among all cancers [1]. PTC has several histopathological variants, including the classical, follicular, tall cell, columnar cells, hobnail, solid, warthin-like, oncocytic, and diffuse sclerosing variants. These variants display distinct growth patterns, cell types, and stromal changes based on biological behaviours, and they are broadly classified into two categories—indolent and aggressive [2,3].

In 1985, Vickery et al. initially described a variant of PTC with the diffuse involvement of the thyroid gland [4]. It was named diffuse sclerosing variant papillary thyroid carcinoma (DSVPTC) by the World Health Organization classification. DSVPTC is characterised by extensive squamous metaplasia, diffuse fibrosis, calcification, abundant lymphocytic infiltration, and psammoma bodies [5].

When compared to classical PTC, DSVPTC is more aggressive, with prominent regional lymph nodes and distant lung metastases during presentation. Moreover, DSVPTC has greater extrathyroidal extension and a higher rate of vascular invasion [6,7,8]. DSVPTC is commonly observed in young patients, with a median age at diagnosis in the third decade of life [5,7,9].

According to the 2015 American Thyroid Association management guidelines for differentiated thyroid carcinoma, DSVPTC has a conflicting prognostic implication and is not considered an aggressive variant of PTC with unfavourable outcomes, such as the tall cell, columnar cell, or hobnail variants [10]. The risk of recurrence is significantly higher in patients with DSVPTC than that in patients with classical PTC but not in those with high-risk PTC [11]. The most frequent genetic aberrations in classical PTC are alterations in the mitogen-activated protein kinase (MAPK) or phosphatidylinositol 3-kinase (PI3K/AKT signal transduction pathways, involving mainly BRAF, RAS, or PIK3CA). Other potent oncogenic drivers in PTCs are RET fusions. There are only a few studies about genetic mutations in DSVPTC [12]. Therefore, we aimed to describe the clinicopathological and genetic characteristics of patients of different ages with DSVPTC.

## 2. Materials and Methods

### 2.1. Patients

This retrospective cohort study included 20,754 patients who underwent initial thyroid surgery between February 2005 and November 2017 at Gangnam Severance Hospital, Yonsei University College of Medicine, Seoul, Republic of Korea. Of these patients, we selected 397 diagnosed with DSVPTC. Histological analysis and confirmation of the diagnosis of DSVPTC were performed by S.-J. Shin. Surgery was performed by different endocrine surgeons of the surgical team of Gangnam Severance Hospital, following the same local regulations and guidelines. A recurrence event was defined as any type of clinical or radiological evidence of recurrence after the initial surgery. In cases with suspicious lesions, recurrence was confirmed by biopsy.

This study was conducted in accordance with the principles of the Declaration of Helsinki of the World Medical Association, Good Clinical Practice, and associated Korean regulations. The requirement for written informed consent was waived owing to the retrospective study design. The study protocol was approved by the Institutional Review Board of Yonsei University (IRB 3-2019-0337), Seoul, Republic of Korea.

### 2.2. Targeted DNA Sequencing and Analysis

Some patients underwent next-generation sequencing (NGS) to explore the effects of genetic variations on DSVPTC. We selected 41 patients through random sampling by their age group and assessed the mutations in 119 genes (Supplementary Table S1). The Baseline characteristics of patients selected for gene sequencing is presented in the Supplementary Table S2.

Genomic DNA was extracted from formalin-fixed paraffin-embedded tumour tissues. We prepared sequencing libraries with Macrogen (Seoul, Republic of Korea) using the SureSelect Target Enrichment Kit (Agilent Technologies, Santa Clara, CA, USA). Two distinct target panels were designed to detect the fusion genes and mutations in the coding exons using the SureSelect Custom DNA Target Enrichment Probes. The libraries were subjected to the Illumina platform in the paired-end (2 × 150 bp) mode. The analytical platforms used by Macrogen included the following: (1) FASTQC, fastp (quality check and trimming); (2) BWA, PICARD, SAMTOOLS, and BEDTOOLS (alignment); and (3) Mutect2 (GATK) and LUMPY (variant calling). We used the human assembly GRCh37/hg19 as the reference genome. The variant call format (VCF version 4.2) provided for each sample by Macrogen was used to identify the variants (annotated with SnpEff version 4.3) in which only the passing variants annotated as PASS were considered the true variants.

Based on the NGS results, we investigated the individual gene mutation patterns of the patients by age group. However, it was necessary to consider the independent effects of individual genes and complex interactions between the biological activities for the mutation affecting the disease. Unexpectedly large biological effects may be involved in diseases upon combining the interactions of several genes and individual mutations. Therefore, we used a gene network to reflect the mutual organic relationships at these biological levels. Through the gene network, we analysed the distribution of the occurrence of genetic variations by age group and the trend of influence propagation by gene interactions.

We constructed the gene network using the Search Tool for the Retrieval of Interacting Genes/Proteins (STRING) database [13]. This database collects and aggregates known and predicted protein–protein interaction information, including physical and functional associations. We obtained the interaction information from the following five major sources: genomic context predictions, high-throughput laboratory experiments, (conserved) co-expression, automated text mining, and previous knowledge from databases. The database provides information on the combined scores of the interactions between genes (or proteins) from various sources, with values ranging from 0 to 1; higher values indicate stronger interactions between two genes.

After constructing the gene network, we examined the effect on DSVPTC through the frequency of mutations and interactions for each group using a machine learning algorithm—the graph-based semi-supervised learning (GSSL) algorithm [14,15]. The GSSL algorithm predicts that nodes with high similarity have similar predictive values for providing the labels of nodes from data expressed in a graphical form (or a network). Labelled and unlabelled data were employed together in the learning and prediction processes, and node classification or the diffusion of information among nodes could be performed through label propagation. For the total n(=l+u) data, in the presence of l labelled data $\left(X_{1},Y_{1}\right),\;\ldots ,\;\left(X_{l},Y_{l}\right)$ and u unlabelled data $\left(X_{l+1},\;0\right),\ldots ,\left(X_{n},\;0\right)$, the optimal solution was obtained by solving the quadratic optimisation problem as follows:

$$\underset{f}{\min}{\left(f-y\right)}^{T}\left(f-y\right)+\mu f^{T}Lf.$$

where y is the label set $y={\left(y_{1},\;\ldots ,\;y_{l},\;0,\;\ldots ,\;0\right)}^{T}$, and the predictive values are $f={\left(f_{1},\;\ldots ,\;f_{l},\;f_{l+1},\;\ldots ,\;f_{n=l+u}\right)}^{T}$. L, the graph Laplacian matrix, is defined as L=D−W, where $D=diag(d_{i})$ and $d_{i}=\sum_{j}w_{ij}$. Moreover, μ is a user-specific hyperparameter that trades off the loss versus smoothness conditions. Eventually, we calculated the GSSL output using the following equation:

$$f={\left(I+\mu L\right)}^{-1}y.$$

We set the frequency of mutations for each group as a label and compared the pattern of influence propagation via the interaction in the gene network using GSSL (by age group). The range of the predicted value f and the output of GSSL were dependent on the range of values of the label in each group. Therefore, the values were scaled through the following equation, which used minimum–maximum normalisation:

$${\tilde{f}}_{i}=\frac{f_{i}-{max}_{f}}{{max}_{f}-{min}_{f}}.$$

### 2.3. Statistical Analyses

All statistical analyses were performed using the Statistical Package for the Social Sciences (SPSS) version 23.0 for Windows (SPSS Inc., Chicago, IL, USA). Categorical data are described as absolute and relative frequencies (percentage), whereas continuous data are presented as the mean and standard deviation. We performed a one-way analysis of variance and the Kruskal–Wallis test for the continuous variables and Pearson’s chi-squared test and Fisher’s exact test for categorical variables. Univariate and multivariate analyses were performed using logistic regression. Statistical significance was set at *p* < 0.05.

## 3. Results

Table 1 summarises the baseline clinical characteristics of 397 patients. The incidence rate of DSVPTC was higher in women (69.5%) than that in men, and the mean age at diagnosis was 36.7 ± 11.6 years. Total thyroidectomy was the most commonly performed surgery (89.7%). We observed recurrence in 46 (11.6%) patients. Regional nodal recurrence (32 [69.6%] patients) was the most common type of recurrence, followed by distant metastasis (7 [15.2%] patients) and operation bed recurrence (6 [13.0%] patients). Central node metastasis was observed in 362 (91.2%) patients, whereas lateral neck metastasis was observed in 237 (59.7%) patients. We divided the patients into six age groups. The group of paediatric and adolescent patients included patients that were 6–20 years old. The oldest patient was 79 years old (Table 2).

Of all the DSVPTC cases, most occurred in patients aged 31–40 years (41.1%). Patients aged ≤20 years displayed the highest incidence (17.3% of all thyroid cancers). There were no differences in capsular invasion, presence of thyroiditis, central node metastasis, or recurrence. Young women (≤20 years, 21–30 years, and 31–40 years) were more affected by DSVPTC. However, the proportions of men and women were relatively even among patients aged ≥41 years. Tumour size was larger in paediatric patients and young adults than in older patients. The rate of lateral neck metastasis was the highest in patients aged ≤20 years (84.6%). V-raf murine sarcoma viral oncogene homolog B1 (*BRAF*) positivity was the highest in patients aged 31–60 years, whereas it was low in young and old patients.

Tumour size, multiplicity, lateral neck metastasis, and *BRAF* positivity were significantly different after dividing the patients into three age groups, as shown in Table 3. The initial presentation of cancer was more aggressive in paediatric and adolescent patients, who demonstrated the largest tumours, most common multiplicity, and more frequent lateral neck metastasis. However, the recurrence rate did not differ among the groups (Table 3).

Of the 119 genes examined, 67 displayed mutations in at least one patient. Table 4 summarises the frequency of gene mutations by age group based on the NGS results. Mutations were seen more frequently in the *BRAF*, Kirsten rat sarcoma virus (*KRAS*), and telomerase reverse transcriptase (*TERT*) genes than in other genes. *BRAF* mutations were particularly prevalent in patients aged 21–60 years (12 out of 19 [63.2%] patients) compared to those seen in patients in other age groups. In contrast, more patients aged ≤20 and ≥61 years displayed *KRAS* and *TERT* mutations than those aged 21–60 years.

**Figure 1:**
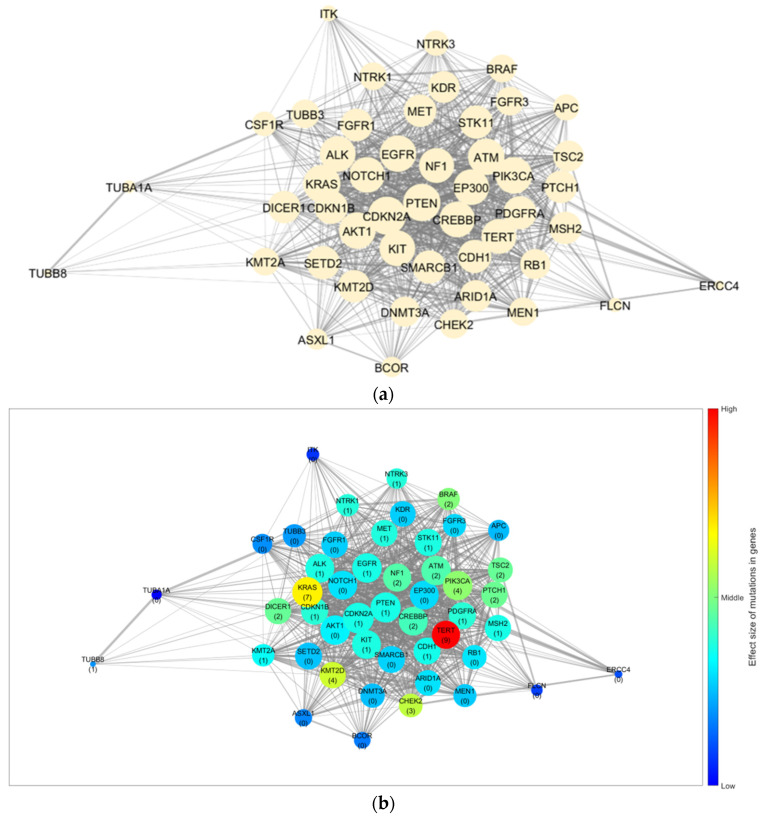

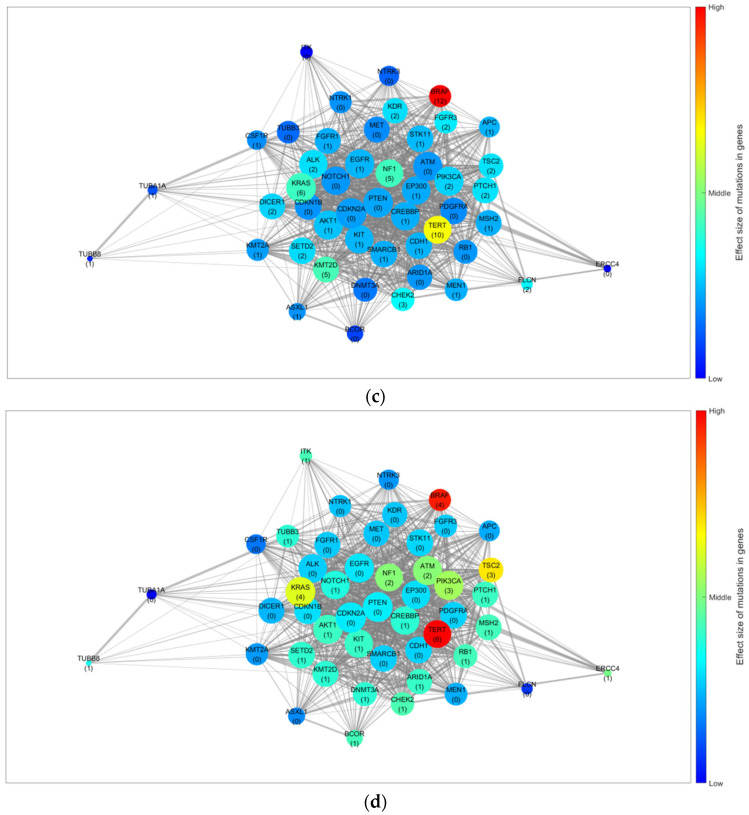
Gene network and results for the trend of influence propagation by age group. (**a**) Base gene network constructed from the NGS results and STRING database. Trends of the effect size of mutations in genes for (**b**) patients aged ≤20, (**c**) 21–60, and (**d**) ≥61 years. The node size indicates the number of linked genes (degree of the node), whereas the edge width indicates the combined score from the STRING database (edge weight). The numbers in parentheses indicate the mutation frequency of the corresponding gene in the group, and the colour of the node indicates the effect size of the mutation and interaction for each gene. STRING, Search Tool for the Retrieval of Interacting Genes/Proteins; NGS, next-generation sequencing.

Using the STRING database, we collected 879 interactions with combined scores among 48 genes (excluding 19 fusion genes from the NGS results) and constructed a gene network. Figure 1a depicts the constructed base gene network with 48 nodes (genes) and 879 edges (interactions).

Figure 1b–d depict the GSSL results by applying the mutation frequency to each group (≤20, 21–60, and ≥61 years) from the base gene network. The patterns of the effect size caused by the genetic mutations differed for each age group. Furthermore, for the quantitative analysis, we selected the top 10 genes based on their calculated effect sizes. Table 5 summarises a comparison of the trends by age group.

*TERT* was identified as the most effective gene in patients aged ≤20 and ≥61 years. However, *BRAF* displayed a marginal difference in value from *TERT* in patients aged ≥61 years, but its effectiveness was significantly lower in those aged ≤20 years. In contrast, *BRAF* was the most effective gene in patients aged 21–60 years. Moreover, the ranking of genes and their order for each group differed. Consequently, we considered *TERT* an effective gene for DSVPTC compared to other genes. However, there were differences in the effect size trends regarding mutations for each group. In addition, by using a gene network rather than comparing the frequency of mutations, we were able to observe cases with a larger effect size owing to the interactions despite small individual frequencies (*CHEK2* in patients aged ≤20 years, *KMT2D* and *NF1* in those aged 21–60 years, and *TSC2* in those aged ≥ 61 years).

## 4. Discussion

Our findings revealed significant age-related differences in DSVPTC. Patients aged ≤20 and ≥61 years displayed significantly different clinicopathological features than those aged 21–60 years. Younger patients had a larger-sized tumour, with frequent multiplicity and lateral neck metastasis. However, the recurrence rates did not differ among the age groups. DSVPTC predominantly affected patients aged 31–40 years, which is consistent with the findings of previous studies [7,9]. However, the mean patient age in the present study was higher than that in a previous study [16].

Tumour size was significantly larger in younger patients and manifested more aggressive patterns, such as more frequent lateral neck metastases, than those seen in older patients. A study comparing DSVPTC and classical PTC in patients with a mean age of 14.3 years demonstrated significantly greater tendencies of DSVPTC toward larger tumour size, disease multifocality, capsular invasion, central and lateral node involvement, and recurrence [17].

Recurrence rates did not differ among age groups. Despite more aggressive features in younger patients, the recurrence rate was not higher than that reported in other age groups. Recurrence occurred in 46 (11.6%) patients, a number lower than that reported by Spinelli et al., who observed that 62% of patients with DSVPTC had recurrent disease [17]. Moreover, despite the more aggressive pathological features and treatments of the microcarcinomas of DSVPTC, there were no differences in the overall survival compared to that of propensity-matched patients with papillary thyroid microcarcinoma [18]. The largest population-level study demonstrated that disease-specific survival was considerably lower in patients with DSVPTC than in those with classical PTC and that DSVPTC diagnosis was an independent factor associated with mortality, with a hazard ratio of 1.8 [8]. In a long-term follow-up study of 19.5 ± 10.6 years, DSVPTC displayed an indolent course, with no cases of death observed during the follow-up period, even in patients with distant metastasis [16].

In the current study, total thyroidectomy (89.7%) followed by radioiodine treatment was more frequently performed than lobectomy (10.3%). Researchers have suggested total thyroidectomy with prophylactic central neck dissection followed by radioiodine treatment as the treatment of choice for DSVPTC when considering the high rates of extrathyroidal extension and lymph node and distant metastases [7]. The risk of persistent/recurrent disease was higher in patients who did not receive routine radioiodine treatment; however, the risk was not significantly different after surgical radioiodine treatment [9]. The lower rate of recurrence in our study may be attributable to the optimal treatment of total thyroidectomy as the first operation followed by radioiodine treatment. A summary of features of tumor aggressiveness in previously published series of patients with DSVPTC are presented in the Supplementary Table S3.

Few studies have reported on genetic alterations in DSVPTC. *BRAF* mutations, rearranged during transfection *RET*/PTC rearrangements, and *ALK* rearrangements are commonly reported mutations [7]. Lim et al. reported that the prevalence of *BRAF* mutations in DSVPTC was significantly lower than that in classical PTC [19]. In contrast, *RET*/PTC rearrangements are major events in DSVPTC, and they are associated with an advanced stage and a higher frequency of persistent disease [20,21]. *RET*/PTC rearrangements are commonly observed in paediatric patients with PTC; therefore, the high incidence of this molecular finding in DSVPTC may be an outcome of patient age [22]. In a previous study, *BRAF* and *RET*/PTC1 rearrangement and *TERT* promoter mutations were associated with more aggressive cancers than malignancies without one of these mutations [23]. Joung et al. identified *RET*/PTC rearrangement and *BRAF* in DSVPTC; however, *NRAS*, *HRAS*, and *KRAS* mutations and paired-box gene 8/peroxisome proliferator-activated receptor g (*PAX8*/*PPARg*) rearrangement were absent [20].

This study has some limitations, including the small sample size, single-institution design, and limitations inherent to the retrospective study design. Further, we did not compare DSVPTC with classical PTC or other aggressive types. Moreover, long-term follow-up is required to evaluate disease recurrence. RET/PTC was not analysed in this study, although it represents one of the most common genetic changes for DSVPTC.

## 5. Conclusions

Patients with DSVPTC exhibit different clinicopathological patterns depending on their age. Despite being frequent and more aggressive in younger patients, DSVPTC also occurs in older patients, showing aggressive behaviour.

## Figures and Tables

**Table 1:** Baseline characteristics of patients with DSVPTC.

Characteristics	N = 397
Female sex	276 (69.5)
Mean age (years)	36.7 ± 11.6
Operation	
Total thyroidectomy	356 (89.7)
Lobectomy	41 (10.3)
Mean follow-up (days)	2865 ± 1088
Recurrence	46 (11.6)
Disease-free survival (days)	1404 ± 1070
Recurrence site	
Operative bed	6 (13.0)
Regional	32 (69.6)
Distant metastasis	7 (15.2)
Pathology	
Cancer size (cm)	1.64 ± 1.13
Multiplicity (bilateral)	157 (39.5)
Capsular invasion	318 (80.1)
Thyroiditis	197 (53.9)
Central node metastasis	362 (91.2)
Lateral neck node metastasis	237 (59.7)
Maximal lymph node metastasis size (cm)	1.29 ± 1.13
*BRAF* positivity	151/257 (58.8)

*BRAF*: V-raf murine sarcoma viral oncogene homolog B1.

**Table 2:** Clinical characteristics across six age groups.

Age Group	≤20 Years	21–30 Years	31–40 Years	41–50 Years	51–60 Years	≥61 Years	*p*-Value
DSVPTC cases (%)	26 (6.5)	85 (21.4)	163 (41.1)	69 (17.4)	40 (10.1)	14 (3.5)	
Thyroid cancer overall	150	1717	6003	6090	4557	2248	
% of total thyroid cancer	17.3	5.0	2.7	1.1	0.9	0.6	
Female	21 (80.8)	70 (82.4)	118 (72.4)	36 (52.2)	23 (57.5)	8 (57.1)	<0.001
Size (cm)	2.69 ± 1.56	1.88 ± 1.30	1.55 ± 0.95	1.38 ± 0.85	1.29 ± 0.91	1.63 ± 1.45	<0.001
Multiplicity (bilateral)	17 (65.4)	27 (31.8)	56 (34.4)	32 (46.4)	16 (40.0)	9 (64.3)	0.034
Capsule invasion	24 (92.3)	71 (83.5)	128 (78.5)	59 (85.5)	26 (65.0)	10 (71.4)	0.052
Thyroiditis	14 (53.8)	45 (52.9)	88 (54.3)	36 (52.2)	23 (57.5)	8 (57.1)	0.996
Central node metastasis	24 (92.3)	79 (92.9)	152 (93.3)	64 (93.3)	32 (80.0)	11 (78.6)	0.064
Lateral neck metastasis	22 (84.6)	53 (62.4)	104 (63.8)	35 (50.7)	16 (40.0)	7 (50.0)	0.004
Maximal lymph node size (cm)	1.82 ± 0.93	1.74 ± 1.57	1.18 ± 0.91	0.91 ± 0.92	1.00 ± 0.92	1.51 ± 1.00	<0.001
*BRAF* positivity	4/15 (26.7)	26/55 (47.3)	72/111 (64.9)	28/43 (65.1)	18/25 (72.0)	3/8 (37.5)	0.01
Recurrence (%)	5 (19.2)	12 (14.1)	18 (11.0)	5 (7.2)	3 (7.5)	3 (21.4)	0.374

*BRAF*: V-raf murine sarcoma viral oncogene homolog B1; DSVPTC: diffuse sclerosing variant papillary thyroid carcinoma.

**Table 3:** Clinical characteristics across three age groups.

Age Group	≤20 Years *n* = 26	21–60 Years *n* = 357	≥61 Years *n* = 14	*p*-Value
Female	21 (80.8)	247 (69.2)	8 (57.1)	0.275
Size (cm)	2.69 ± 1.56	1.56 ± 1.04	1.63 ± 1.45	<0.001
Multiplicity (bilateral)	17 (65.4)	131 (36.7)	9 (64.3)	0.007
Capsule invasion	24 (92.3)	284 (79.6)	10 (71.4)	0.206
Thyroiditis	14 (53.8)	192 (53.9)	8 (57.1)	0.972
Central node metastasis	24 (92.3)	327 (91.6)	11 (78.6)	0.236
Lateral neck metastasis	22 (84.6)	208 (58.3)	7 (50.0)	0.023
Maximal lymph node size (cm)	1.82 ± 0.93	1.24 ± 1.14	1.51 ± 1.00	0.083
*BRAF* positivity	4/15 (26.7)	144/234 (61.5)	3/8 (37.5)	0.013
Recurrence (%)	5 (19.2)	38 (10.6)	3 (21.4)	0.113

*BRAF*: V-raf murine sarcoma viral oncogene homolog B1.

**Table 4:** Results of next-generation sequencing: number of patients with gene mutations by age group.

Gene	Group			Gene	Group			Gene	Group		
	≤ 20 Years (*n* = 13)	21–60 Years (*n* = 19)	≥ 61 Years (*n* = 9)		≤ 20 Years (*n* = 13)	21–60 Years (*n* = 19)	≥ 61 Years (*n* = 9)		≤ 20 Years (*n* = 13)	21–60 Years (*n* = 19)	≥ 61 Years (*n* = 9)
*AKT1*	0 (0.0%)	1 (5.3%)	1 (11.1%)	*EGFR*	1 (7.7%)	1 (5.3%)	0 (0.0%)	*NTRK1*	1 (7.7%)	0 (0.0%)	0 (0.0%)
*ALK*	1 (7.7%)	2 (10.5%)	0 (0.0%)	*EML4-NTRK3*	0 (0.0%)	1 (5.3%)	0 (0.0%)	*NTRK3*	1 (7.7%)	0 (0.0%)	0 (0.0%)
*ALK-GALNT14*	2 (15.4%)	0 (0.0%)	1 (11.1%)	*EP300*	0 (0.0%)	1 (5.3%)	0 (0.0%)	*NUP210-PPARG*	0 (0.0%)	0 (0.0%)	1 (11.1%)
*ALK-MSN*	0 (0.0%)	1 (5.3%)	0 (0.0%)	*ERCC4*	0 (0.0%)	0 (0.0%)	1 (11.1%)	*PDGFRA*	1 (7.7%)	0 (0.0%)	0 (0.0%)
*ALK-NPM1*	0 (0.0%)	0 (0.0%)	1 (11.1%)	*ETV6-NTRK3*	1 (7.7%)	0 (0.0%)	1 (11.1%)	*PIK3CA*	4 (30.8%)	2 (10.5%)	3 (33.3%)
*APC*	0 (0.0%)	1 (5.3%)	0 (0.0%)	*FGFR1*	0 (0.0%)	1 (5.3%)	0 (0.0%)	*PTCH1*	2 (15.4%)	2 (10.5%)	1 (11.1%)
*ARID1A*	0 (0.0%)	0 (0.0%)	1 (11.1%)	*FGFR3*	0 (0.0%)	2 (10.5%)	0 (0.0%)	*PTEN*	1 (7.7%)	0 (0.0%)	0 (0.0%)
*ASXL1*	0 (0.0%)	1 (5.3%)	0 (0.0%)	*FLCN*	0 (0.0%)	2 (10.5%)	0 (0.0%)	*RB1*	0 (0.0%)	0 (0.0%)	1 (11.1%)
*ATM*	2 (15.4%)	0 (0.0%)	2 (22.2%)	*FN1-ALK*	1 (7.7%)	0 (0.0%)	0 (0.0%)	*SETD2*	0 (0.0%)	2 (10.5%)	1 (11.1%)
*BCOR*	0 (0.0%)	0 (0.0%)	1 (11.1%)	*ITK*	0 (0.0%)	0 (0.0%)	1 (11.1%)	*SMARCB1*	0 (0.0%)	1 (5.3%)	0 (0.0%)
*BRAF*	2 (15.4%)	12 (63.2%)	4 (44.4%)	*KDR*	0 (0.0%)	2 (10.5%)	0 (0.0%)	*SPTBN1-ALK*	3 (23.1%)	0 (0.0%)	1 (11.1%)
*BRAF-SND1*	1 (7.7%)	1 (5.3%)	0 (0.0%)	*KIT*	1 (7.7%)	1 (5.3%)	1 (11.1%)	*STK11*	1 (7.7%)	1 (5.3%)	0 (0.0%)
*BRAF-SUGCT*	1 (7.7%)	0 (0.0%)	0 (0.0%)	*KMT2A*	1 (7.7%)	1 (5.3%)	0 (0.0%)	*STRN-ALK*	1 (7.7%)	0 (0.0%)	0 (0.0%)
*CCDC6-RET*	3 (23.1%)	2 (10.5%)	0 (0.0%)	*KMT2D*	4 (30.8%)	5 (26.3%)	1 (11.1%)	*TERT*	9 (69.2%)	10 (52.6%)	6 (66.7%)
*CDH1*	1 (7.7%)	1 (5.3%)	0 (0.0%)	*KRAS*	7 (53.9%)	6 (31.6%)	4 (44.4%)	*TP53-DNAH2*	0 (0.0%)	1 (5.3%)	0 (0.0%)
*CDKN1B*	1 (7.7%)	0 (0.0%)	0 (0.0%)	*LMNA-ALK*	0 (0.0%)	1 (5.3%)	0 (0.0%)	*TRIO-TERT*	0 (0.0%)	0 (0.0%)	1 (11.1%)
*CDKN2A*	1 (7.7%)	0 (0.0%)	0 (0.0%)	*MACF1-BRAF*	0 (0.0%)	0 (0.0%)	2 (22.2%)	*TSC2*	2 (15.4%)	2 (10.5%)	3 (33.3%)
*CHEK2*	3 (23.1%)	3 (15.8%)	1 (11.1%)	*MEN1*	0 (0.0%)	1 (5.3%)	0 (0.0%)	*TUBA1A*	0 (0.0%)	1 (5.3%)	0 (0.0%)
*CLIP4-ALK*	1 (7.7%)	0 (0.0%)	0 (0.0%)	*MET*	1 (7.7%)	0 (0.0%)	0 (0.0%)	*TUBB3*	0 (0.0%)	0 (0.0%)	1 (11.1%)
*CREBBP*	2 (15.4%)	1 (5.3%)	1 (11.1%)	*MKRN1-BRAF*	0 (0.0%)	1 (5.3%)	0 (0.0%)	*TUBB8*	1 (7.7%)	1 (5.3%)	1 (11.1%)
*CSF1R*	0 (0.0%)	1 (5.3%)	0 (0.0%)	*MSH2*	1 (7.7%)	1 (5.3%)	1 (11.1%)	*VCL-ALK*	0 (0.0%)	0 (0.0%)	1 (11.1%)
*DICER1*	2 (15.4%)	2 (10.5%)	0 (0.0%)	*NF1*	2 (15.4%)	5 (26.3%)	2 (22.2%)				
*DNMT3A*	0 (0.0%)	0 (0.0%)	1 (11.1%)	*NOTCH1*	0 (0.0%)	0 (0.0%)	1 (11.1%)				

*AKT1*, AKT serine/threonine kinase 1; *ALK*, anaplastic lymphoma kinase; *GALNT14*, N-acetylgalactosaminyltransferase 14; *MSN*, moesin; *NPM1*, nucleophosmin; *APC*, adenomatous polyposis coli; *ARID1A*, AT-rich interaction domain 1A; *ASXL1*, ASXL transcriptional regulator 1; *ATM*, ataxia telangiectasia mutated; *BCOR*, BCL6 corepressor; *BRAF*, V-raf murine sarcoma viral oncogene homolog B1; *SND1*, Staphylococcal nuclease and Tudor domain containing 1; *SUGCT*, succinyl-CoA:glutarate-CoA transferase; *CCDC6-RET*, coiled coil domain containing 6-rearranged during transfection; *CDH1*, cadherin 1; *CDKN1B*, cyclin-dependent kinase inhibitor 1B; *CDKN2A*, cyclin-dependent kinase inhibitor 2A; *CHEK2*, checkpoint kinase 2; *CLIP4-ALK*, CAP-Gly domain containing linker protein family member 4-anaplastic lymphoma kinase; *CREBBP*, cyclic adenosine monophosphate response element binding protein binding protein; *CSF1R*, colony stimulating factor 1 receptor; *DICER1*, dicer 1, ribonuclease III; *DNMT3A*, DNA methyltransferase 3 alpha; *EGFR*, epidermal growth factor receptor; *EML4-NTRK3*, EMAP-like 4-neurotrophic receptor tyrosine kinase 3; *EP300*, E1A-associated protein p300; *ERCC4*, ERCC excision repair 4, endonuclease catalytic subunit; *ETV6-NTRK3*, translocation-Ets-leukaemia virus-neurotrophic receptor tyrosine kinase 3; *FGFR1*, fibroblast growth factor receptor 1; *FGFR3*, fibroblast growth factor receptor 3, *FLCN*, folliculin; *FN1-ALK*, fibronectin 1-anaplastic lymphoma kinase; *ITK*, IL2 inducible T cell kinase; *KDR*, kinase insert domain receptor; *KIT*, KIT proto-oncogene, receptor tyrosine kinase; *KMT2A*, lysine methyltransferase 2A; *KMT2D*, lysine methyltransferase 2D; *KRAS*, Kirsten rat sarcoma virus; *LMNA-ALK*, lamin A/C-anaplastic lymphoma kinase; *MACF1-BRAF*, microtubule actin crosslinking factor 1-V-raf murine sarcoma viral oncogene homolog B1; *MEN1*, multiple endocrine neoplasia type 1; *MET*, MET proto-oncogene, receptor tyrosine kinase; *MKRN1-BRAF*, Makorin ring finger protein-1-V-raf murine sarcoma viral oncogene homolog B1; *MSH2*, MutS homolog 2; *NF1*, neurofibromin 1; *NOTCH1*, neurogenic locus notch homolog protein 1; *NTRK1*, neurotrophic receptor tyrosine kinase 1; *NTRK3*, neurotrophic receptor tyrosine kinase 3; *NUP210-PPARG*, nucleoporin 210-peroxisome proliferator-activated receptor gamma; *PDGFRA*, platelet-derived growth factor receptor alpha; *PIK3CA*, phosphatidylinositol-4,5-bisphosphate 3-kinase catalytic subunit alpha; *PTCH1*, protein patched homolog 1; *PTEN*, phosphatase and TENsin homolog deleted on chromosome 10; *RB1*, retinoblastoma protein; *SETD2*, SET domain containing 2, histone lysine methyltransferase; *SMARCB1*, SWI/SNF related, matrix associated, actin dependent regulator of chromatin, subfamily B, member 1; *SPTBN1-ALK*, spectrin beta, non-erythrocytic 1-anaplastic lymphoma kinase; *STK11*, serine/threonine kinase 11; *STRN-ALK*, striatin-anaplastic lymphoma kinase; *TERT*, telomerase reverse transcriptase; *TP53-DNAH2*, tumour protein p53-DNA replication helicase/nuclease 2; *TRIO-TERT*, trio rho guanine nucleotide exchange factor-telomerase reverse transcriptase; *TSC2*, tuberous sclerosis complex 2; tubulin alpha 1a; *TUBB3*, tubulin beta 3 class III; *TUBB8*, tubulin beta 3 class VIII; *VCL-ALK*, vinculin-anaplastic lymphoma kinase.

**Table 5:** Comparison of the trends in effective genes by age group.

Rank	Group								
	≤20 Years (*n* = 13)			21–60 Years (*n* = 19)			≥61 Years (*n* = 9)		
	Gene	$\tilde{f}$	$N_{mut}$	Gene	$\tilde{f}$	$N_{mut}$	Gene	$\tilde{f}$	$N_{mut}$
1	*TERT*	1.000	9 (69.2%)	*BRAF*	1.000	12 (63.2%)	*TERT*	1.000	6 (66.7%)
2	*KRAS*	0.679	7 (53.8%)	*TERT*	0.657	10 (52.6%)	*BRAF*	0.961	4 (44.4%)
3	*KMT2D*	0.604	4 (30.8%)	*KMT2D*	0.426	5 (26.3%)	*TSC2*	0.695	3 (33.3%)
4	*CHEK2*	0.584	3 (23.1%)	*NF1*	0.415	5 (26.3%)	*KRAS*	0.626	4 (44.4%)
5	*PIK3CA*	0.521	4 (30.8%)	*KRAS*	0.411	6 (31.6%)	*PIK3CA*	0.547	3 (33.3%)
6	*BRAF*	0.506	2 (15.4%)	*CHEK2*	0.341	3 (15.8%)	*NF1*	0.508	2 (22.2%)
7	*PTCH1*	0.475	2 (15.4%)	*FLCN*	0.325	2 (10.5%)	*ATM*	0.508	2 (22.2%)
8	*DICER1*	0.469	2 (15.4%)	*FGFR3*	0.311	2 (10.5%)	*ERCC4*	0.487	1 (11.1%)
9	*TSC2*	0.468	2 (15.4%)	*TSC2*	0.300	2 (10.5%)	*CHEK2*	0.431	1 (11.1%)
10	*NF1*	0.441	2 (15.4%)	*PTCH1*	0.298	2 (10.5%)	*ITK*	0.426	1 (11.1%)

$N_{mut}$ refers to the number of patients with genetic mutations and their proportion in the group. *TERT*, telomerase reverse transcriptase; *KRAS*, Kirsten rat sarcoma virus; *KMT2D*, lysine methyltransferase 2D; *CHEK2*, checkpoint kinase 2; *PIK3CA*, phosphatidylinositol-4,5-bisphosphate 3-kinase catalytic subunit alpha; *BRAF*, V-raf murine sarcoma viral oncogene homolog B1; *PTCH1*, protein patched homolog 1; *DICER1*, dicer 1, ribonuclease III; *NF1*, neurofibromin 1; *TSC2*, tuberous sclerosis complex 2; *FLCN*, folliculin; *FGFR3*, fibroblast growth factor receptor 3; *ATM*, ataxia telangiectasia mutated; *ERCC4*, ERCC excision repair 4, endonuclease catalytic subunit; *ITK*, IL2 inducible T cell kinase.

## Data Availability

Data are available upon request to the author.

## Supplementary Materials

The following supporting information can be downloaded at: https://www.mdpi.com/article/10.3390/cancers15123101/s1, Table S1: List of genes and gene fusions analyzed through next-generation sequencing; Table S2: Baseline characteristics of patients selected for gene sequencing; Table S3: Feature of tumor aggressiveness in previously published series of patients with DSVPTC [7,8,16,17].
