# Supplementary material for: Clinicopathological and Genetic Characteristics of Patients of Different Ages with Diffuse Sclerosing Variant Papillary Thyroid Carcinoma
Source: Cancers (Basel). 2023 Jun 7;15(12):3101. doi: 10.3390/cancers15123101 (PMC10296723; doi:10.3390/cancers15123101)
Supplement: Supplementary file 1 [file cancers-15-03101-s001.zip › cancers-2417100-supplementary.pdf]

Table S1. List of genes and gene fusions analyzed through next-generation sequencing.

AKT1
APC
ARID1A
ARID2
ASXL1
ATM
ATRX
AXIN1
BCOR
BCORL1
BRAF
CCDC6
CD274
CDH1
CDKN1B
CDKN2A
CDKN2C
CHD4
CHEK2
CREBBP
CSF1R
CTNNA2
DICER1
DNMT3A
EGFR
EIF1AX
EP300
ERCC4
FGFR1
FGFR3
FLCN
FLT1
FLT4
FN1
GRIN2A
HRAS
IDH1
ITK
KDR

KIT
KMT2A
KMT2D
KRAS
LATS1
LATS2
LCK
MAP2K1
MAPK1
MAPK3
MEN1
MET
MSH2
MTOR
NF1
NF2
NOTCH1
NRAS
PBRM1
PDCD1
PDGFRA
PDGFRB
PIK3CA
PPARGC1A
PTCH1
PTEN
RB1
RBM10
RET
RNF43
SETD2
SMARCB1
SNAI1
SPOP
STK11
STRN
TET2
THADA
TOP2A
TSC2

TUBA1A
TUBA1B
TUBA1C
TUBA3C
TUBA3E
TUBA4A
TUBB
TUBB1
TUBB2A
TUBB2B
TUBB3
TUBB4A
TUBB4B
TUBB6
TUBB8
VIM
ZEB1
TERT
ETV6-NTRK3
CCDC6-RET
CLIP4-ALK
SPTBN1-ALK
STRN-ALK
ALK-GALNT14
NTRK1
NTRK3
TP53-DNAH2
BRAF-SND1
ALK-MSN
MACF1-BRAF
FN1-ALK
EML4-NTRK3
TRIO-TERT
ALK
NUP210-PPARG
MKRN1-BRAF
LMNA-ALK
ALK-NPM1
VCL-ALK

BRAF-SUGCT

Table S2. Baseline characteristics of patients selected for gene sequencing.

**Characteristics**	**N = 41**
Female sex	28 (68.3)
Mean age (years)	39.3 ± 21.1
Operation	
Total thyroidectomy	39 (95.1)
Lobectomy	2 (4.9)
Mean follow-up (days)	2865 ± 1088
Recurrence	46 (11.6)
Disease free survival (days)	2561 ± 1083
Recurrence site	
Operative bed	0 (0)
Regional	5 (83.3)
Distant metastasis	1 (16.7)
Pathology	
Cancer size (cm)	2.29 ± 1.62
Multiplicity (bilateral)	21 (51.2)
Capsular invasion	35 (85.4)
Thyroiditis	23 (56.1)
Central node metastasis	37 (90.2)
Lateral neck node metastasis	31 (75.6)
Maximal lymph node metastasis size (cm)	1.67 ± 1.08
BRAF positivity	14/30 (46.7)

Table S3. Feature of tumor aggressiveness in previously published series of patients with DSVPTC.

Features of tumor aggressiveness	Ratio of expected events (%)
Tumor size [cm]	1.80[1], 1.70[2]; 4.2[3]
Multifocality	35.8[1]; 29.5[2]; 72.7[4]; 72.0[3]
Lymphovascular invasion	38.6[1]; 48.3[4]
Extrathyroidal extension	50.4[1]; 31.0[2]; 51.7[4]; 76[3]
Lymph node metastasis	80.3[1]; 72.2[2]; 81.8[4]; 92[3]
Distant metastasis	11.6[1]; 7.3[2]
Recurrence	22.0[1]; 62[3]

Reference

1. Vuong, H.G.; Kondo, T.; Pham, T.Q.; Oishi, N.; Mochizuki, K.; Nakazawa, T.; Hassell, L.; Katoh, R. Prognostic significance of diffuse sclerosing variant papillary thyroid carcinoma: a systematic review and meta-analysis. *Eur J Endocrinol* **2017**, *176*, 433-441, doi:10.1530/EJE-16-0863.
2. Kazaure, H.S.; Roman, S.A.; Sosa, J.A. Aggressive variants of papillary thyroid cancer: incidence, characteristics and predictors of survival among 43,738 patients. *Ann Surg Oncol* **2012**, *19*, 1874-1880, doi:10.1245/s10434-011-2129-x.
3. Spinelli, C.; Strambi, S.; Bakkar, S.; Nosiglia, A.; Elia, G.; Bertocchini, A.; Calani, C.; Leoni, M.;

Morganti, R.; Materazzi, G. Surgical Management of Diffuse Sclerosing Variant of Papillary Thyroid Carcinoma. Experience in 25 Patients. *World J Surg* **2020**, *44*, 155-162, doi:10.1007/s00268-019-05230-5.

4. Cavaco, D.; Martins, A.F.; Cabrera, R.; Vilar, H.; Leite, V. Diffuse sclerosing variant of papillary thyroid carcinoma: outcomes of 33 cases. *Eur Thyroid J* **2022**, *11*, doi:10.1530/ETJ-21-0020.
